# Annulus Fibrosus Cell Characteristics Are a Potential Source of Intervertebral Disc Pathogenesis

**DOI:** 10.1371/journal.pone.0096519

**Published:** 2014-05-05

**Authors:** Li Jin, Qihai Liu, Phillip Scott, Dawei Zhang, Francis Shen, Gary Balian, Xudong Li

**Affiliations:** Department of Orthopaedic Surgery, University of Virginia, Charlottesville, Virginia, United States of America; Faculté de médecine de Nantes, France

## Abstract

In the end stage of intervertebral disc degeneration, cartilage, bone, endothelial cells, and neurons appear in association with the worsening condition. The origin of the abnormal cells is not clear. This study investigated the properties of progenitor cells in the annulus fibrosus (AF) using one *in vitro* and two *in vivo* models. Cultivation of rabbit AF cells with chondrogenic media significantly increased expressions of collagen and aggrecan. Upon exposure to osteogenic conditions, the cultures showed increased mineralization and expression of osteopontin, runx2, and bmp2 genes. Two models were used in the *in vivo* subcutaneous implantation experiments: 1) rabbit AF tissue in a demineralized bone matrix (DBM) cylinder (DBM/AF), and, 2) rat intact and needle punctured lumbar discs. Bone formation in the AF tissue was detected and hypertrophic chondrocytes and osteoblasts were present 1 month after implantation of the DBM/AF to nude mice. In addition to collagen I and II, immunostaining shows collagen X and osteocalcin expression in DBM/AF specimens 4 months after implantation. Similar changes were detected in the injured discs. Almost the entire needle punctured disc had ossified at 6 months. The results suggest that AF cells have characteristics of progenitor cells and, under appropriate stimuli, are capable of differentiating into chondrocytes and osteoblasts *in vitro* as well as *in vivo*. Importantly, these cells may be a target for biological treatment of disc degeneration.

## Introduction

Degenerative disc disease affects the majority of the population and treatment costs up to 100 billion dollars annually [1], [2]. Current methods of treatment include non-surgical therapies such as physical therapy, spinal fusion, and disc replacement surgeries [3],[4]. However, none of these methods restore full spine structure or function. This is due in part to the underlying mechanisms and pathologies of the disease being largely unknown. A better understanding of intervertebral disc (IVD) cell biology is likely to prevent onset of the disease and may help develop effective treatment strategies.

Intervertebral discs, located between adjacent vertebrae, are responsible for relieving mechanical and compression stress. The IVD comprises three distinct regions: nucleus pulposus (NP), annulus fibrosus (AF), and endplates. Each of these regions is structurally and biochemically different, but function in concert to maintain the structural integrity of the spine. This is achieved through the disc's network of various structural components including types of collagen, aggrecan, and elastin fibers that work to maintain hydrostatic pressure to relieve the effects of compression on the spine [5], [6]. Over time, focal biochemical and biomechanical changes diminish the ability of IVDs to replenish its matrix constituents, and, consequently, the disc functions less efficiently. The process can be exacerbated by injury, aging or overuse [7]. The etiology of disc degeneration is multifactorial, with no clear biological causes [8], [9].

In the later stages of disease, many different cell types are found in the disc including chondrocytes, osteoblasts, and neurons, but it is not known how changes in cellularity occur [10]–[12]. The source of these different cell types in the degenerated IVD is controversial. One hypothesis suggests that foreign cell types migrate into the degenerating IVD from surrounding spinal tissue [13], [14], while another promotes the notion that the disc contains progenitor cells that differentiate into these different cell types [15]. Various studies have shown that IVD contains cells that can differentiate into multiple cell lineages. In the degenerative IVD, hypertrophic chondrocyte differentiation and calcification are observed. Abundant expressions of collagen type X, osteoprotegerin, Runx2 occur in the degenerate discs [16]. Risbud *et al*. showed the presence of skeletal progenitor cells in the degenerated human intervertebral disc [17]. The mesenchymal stem cell markers have been found in the NP and AF of degenerated discs [18], [19]. In a pig annular injury model, Mizrahi *et al*. demonstrated that both healthy and degenerated disc cells are able to differentiate into mesenchymal lineages [20]. Using different protocols that induce cell differentiation, we demonstrated that, upon appropriate stimulation, human AF cells can differentiate into adipocytes, chondrocytes, neurons, osteoblasts, and endothelial cells [15]. Similarly, a few studies have shown that the NP contains progenitor cells [17], [21]. The effect of the progenitor cells in disc degeneration is not understood. However, all these studies were *in vitro* experiments. There have been no *in vivo* experiments thus far.

The goal of this study is to further characterize the progenitor property of the inner AF cell in both *in vitro* and *in vivo* models. Our hypothesis is that the inner AF tissue could develop into cartilage-like or bone tissue upon biomechanical or biochemical stimuli *in vivo*. Two models were used in the *in vivo* subcutaneous implantation experiments: 1) Rabbit AF tissue in a DBM cylinder, which provides an osteogenic biochemical stimulus. 2) Rat needle punctured lumbar discs, which provides a biomechanical stimuli both from unloading and AF injury.

## Materials and Methods

The study was carried out in strict accordance with the recommendations in the Guide for the Care and Use of Laboratory Animals of National Institutes of Health. All animal procedures were performed according to protocols approved by the Animal Care and Use Committee at the University of Virginia (Permit Number: 3534). All surgery was performed under anesthesia to ameliorate suffering.

### Cell Isolation and Culture

Inner AF cells were isolated from New Zealand white rabbits as reported previously [15]. Briefly, after euthanasia, the spine was exposed and inner AF tissues were harvested from the L2-L4 lumbar IVD. The inner AF tissues were cut into small pieces and digested with 0.01% collagenase (Serva, Germany) at 37°C for 2–4 hours with mild agitation. The cells were pelleted by centrifugation at 500 g for 10 minutes and suspended in culture medium (DMEM, 10% FBS, 1% Penicillin/streptomycin). Cells were cultured at 37°C in a humidified atmosphere of 95% air and 5% CO_2_. Culture medium was changed every three days. Full population of rabbit AF cells at passage 2–4 were used for the later experiments. The *in vitro* experiments were performed three times in triplicate and inner AF cells from three rabbits were used.

### Osteogenic Differentiation

Osteogenic differentiation was performed according our published protocol [15], [22]. Rabbit AF cells were plated onto 24-well culture plates at a density of 5×10^4^ cells/cm^2^. The monolayer cells were grown up to 100% confluence and the culture media were replaced with osteogenic induction media (DMEM supplemented with 0.01 µM 1,25-dihydroxyvitamin D3 (R & D Systems, MN), 50 µM ascorbate-2-phosphate (Sigma, MO), and 10 mM β-glycerophosphate (Sigma, MO) for 4 weeks. Cells cultured in growth medium (DMEM with 10% FBS) were used as controls.

### Chondrogenic Differentiation

For chondrogenic differentiation, rabbit AF cells were cultured in a pellet culture system as previously described [15]. AF cells (2×10^5^) were pelleted by gentle centrifugation for 5 minutes at 500 g in a 15-mL polypropylene tube. The pellets were then cultured in chondrogenic medium for three weeks. Chondrogenic induction medium consisted of DMEM supplemented with 1% fetal bovine serum, 10 nM dexamethasone, 10 ng/ml transforming growth factor β1 (BD Biosciences, NJ), 1% ITS-Premix (6.25 g/ml insulin, 6.25 g/ml transferrin, 6.25 ng/ml selenium acid, 1.25 mg/ml bovine serum albumin, and 5.35 mg/ml linoleic acid (Collaborative Biomedical, MA), and 37.5 g/ml ascorbic-2-phosphate (Sigma, MO). Cell pellets cultured in DMEM with 1% fetal bovine serum and 1% ITS-Premix were used as controls.

### Real-Time RT-PCR

Total RNA was extracted with Trizol reagent and cDNA was generated with iScript cDNA synthesis kit (Bio-Rad, CA) following the manufacture's instruction. Quantitative RT-PCR was performed with an iQ 5 multicolor real-time PCR Detection System (Bio-Rad, CA) using QuantiTect SYBR Green PCR kit (Qiagen, CA). The expression of the target gene was normalized to the 18 s expression. Primer sequences are shown in Table1.

**Table 1:** Primer Sequences for real-time PCR.

Gene	Primers	Accession Numbers
18 s	F: 5′-CGGCGACGACCCATTCGAAC-3′	NR_003278
	R: 5′-GAATCGAACCCTGATTCCCCGTC-3′	
Aggrecan	F: 5′-AGGATGGCTTCCACCAGTGC-3′	L38480
	R: 5′-TGCGTAAAAGACCTCACCCTCC-3′	
Collagen II	F: 5′-ACAGCAGGTTCACCTATAC-3′	NM_001195671
	R: 5′-CCCACTTACCGGTGTT-3′	
Runx2	F: 5′-TGATGACACTGCCACCTCTG-3′	AY598934
	R: 5-GGGATGAAATGCTTGGGAACTG-3′	
BMP2	F: 5′-AACACAAACAGCGGAAAC-3′	NM_001082650
	R: 5′-ATGGTTAGTGGAGTTCAGG-3′	
Osteopontin	F: 5′-CACCATGAGAATCGCCGT-3′	D11411
	R: 5′-CGTGACTTTGGGTTTCTACGC-3′	

### Alizarin Red S Staining

After osteogenic induction, cells were fixed with 5% formalin for 15 minutes followed by incubation with 0.2% (W/V) Alizarin Red S Solution (Chemicon International, CA) for 10 minutes at room temperature. The cell layers were washed several times with distilled water and viewed under a light microscope. For quantification, calcium deposition was washed intensively with deionized water to remove the unbound dye, and then dissolved with cetylpyridinium chloride overnight. Absorbance of the extracts was measured on a spectrophotometer at 550 nm in a 96-well plate.

### Safranin-O Staining

Pellets were fixed with 4% paraformaldehyde for 4 hours and embedded in paraffin. Sections in 5 µm thickness were stained with 0.1% Safranin-O for detection of proteoglycan [23], [24].

### Glycosaminoglycan Assay

The content of glycosaminoglycan (GAG) was measured as described previously [25]. Amino sugars, as a measure of GAG, were determined using a dimethylmethylene blue colorimetric assay (Cresent Chemicals Corp, NY) with chondrotin sulfate-C (Sigma, MO) as a standard. DNA content was analyzed using the Hoechst 33258 dye (Sigma, MO) assay with a calf thymus DNA standard. The content was normalized to DNA content.

### Demineralized Bone Matrix/Annulus Tissue Constructs Preparation

Demineralized bone matrix (DBM) was prepared from forearm bones of New Zealand white rabbits following our published protocol [26], [27]. Briefly, bones were extracted with a 1∶1 mixture of chloroform and methanol (30 ml/g of bone) for 1.5 h, and then subjected to the following steps: Demineralized at 2°C with 0.6 M hydrochloric acid (60 mg/g) overnight; Washed with sterile deionized water until pH 7.4, 2 M CaCl_2_ for 1 h at 2°C, 0.5 M EDTA for 1 h followed by 8 M LiCl for 1 h; Finally washed with deionized water at 55°C. The DBM was then incubated with DMEM for 1 h. AF tissue was dissected from rabbits and removed from connective tissue and end plates. The DBM/AF constructs were fabricated by inserting the AF tissue into the DBM cylinders. To avoid the variance of DBM osteogenic potential, DBMs from the same rabbit forearms were prepared for the experimental groups at the same time point.

### Herniated Disc Preparation

The lumber IVDs from adult male Sprague-Dawley rats (150–175 g) were dissected from connective tissues under sterile conditions. The intact discs were composed of endplates, AF and NP tissues. For disc herniation injury, the intact discs were punctured with a 27G needle. Discs from either the intact or the injured were used for *in vivo* implantation.

### Subcutaneous Implantation of IVD

Ten week-old female athymic mice (NCr-nu/nu, The Jackson's laboratory, ME) were used for the study. All the animals received intraperitoneal injections of Ketamine/Xylazine before surgery. The intact discs, injured discs, AF, DBM, or the DBM/AF constructs were then subcutaneously implanted in the dorsal space of the nude mice. Briefly, a 10-mm incision was cut in the dorsal skin of mice between the scapulare using a size 15 scalpel blade, and a disc or DBM/AF construct was embedded into each pocket. The AF and DBM/AF specimens were harvested at 4 and 12 weeks after implantation and injured or intact IVD specimens were harvested at 1, 2, 4 and 6 months after implantation. All specimens were fixed with 4% paraformaldehyde. Each group has 4 specimen at each time point.

### Two-dimensional Radiographs

At 4 and 8 weeks after implantation, 2D radiographs of the specimen were taken to qualitatively assess bone formation (n = 4).

### Histological and Immunostaining

The specimens (n = 4) were embedded into paraffin and sectioned at 5 µm. The sections were subjected to standard Safranin-O staining as previously described [28]. Immunostaining of Collagen I, II, X, and osteocalcin was performed as previously described [28]. Primary antibodies used were: Biotinlyated collagen I and II antibodies (1∶400, Chondrex, WA), mouse collagen X antibody (1∶500, Sigma, MO), mouse osteocalcin antibody (1∶500, Abcam, MA). For collagen X and osteocalcin detection, biotinylated rabbit anti-mouse secondary antibody (1∶1000, Dako, CA) was used. The sections with the same procedure but omitting the primary antibody were used as a negative control.

### Statistical Analysis

All experiments were performed in triplicate and data were presented as mean ± standard deviation (SD). For the in vivo experiments, with a standard deviation of 0.25, a sample size of 4 would result in a statistical power of 0.8. Thus, we used 4 samples for each group at each time point. Statistical analyses for quantitative assays were performed by One-Way ANOVA using Microsoft Excel 2010 software. The Shaprio-Wilk test was used to test the normality. A *p*-value less than 0.05 was considered statistically significant.

## Results

### Chondrogenic Potential of Annulus Cells in 3D Culture

**Figure 1:**
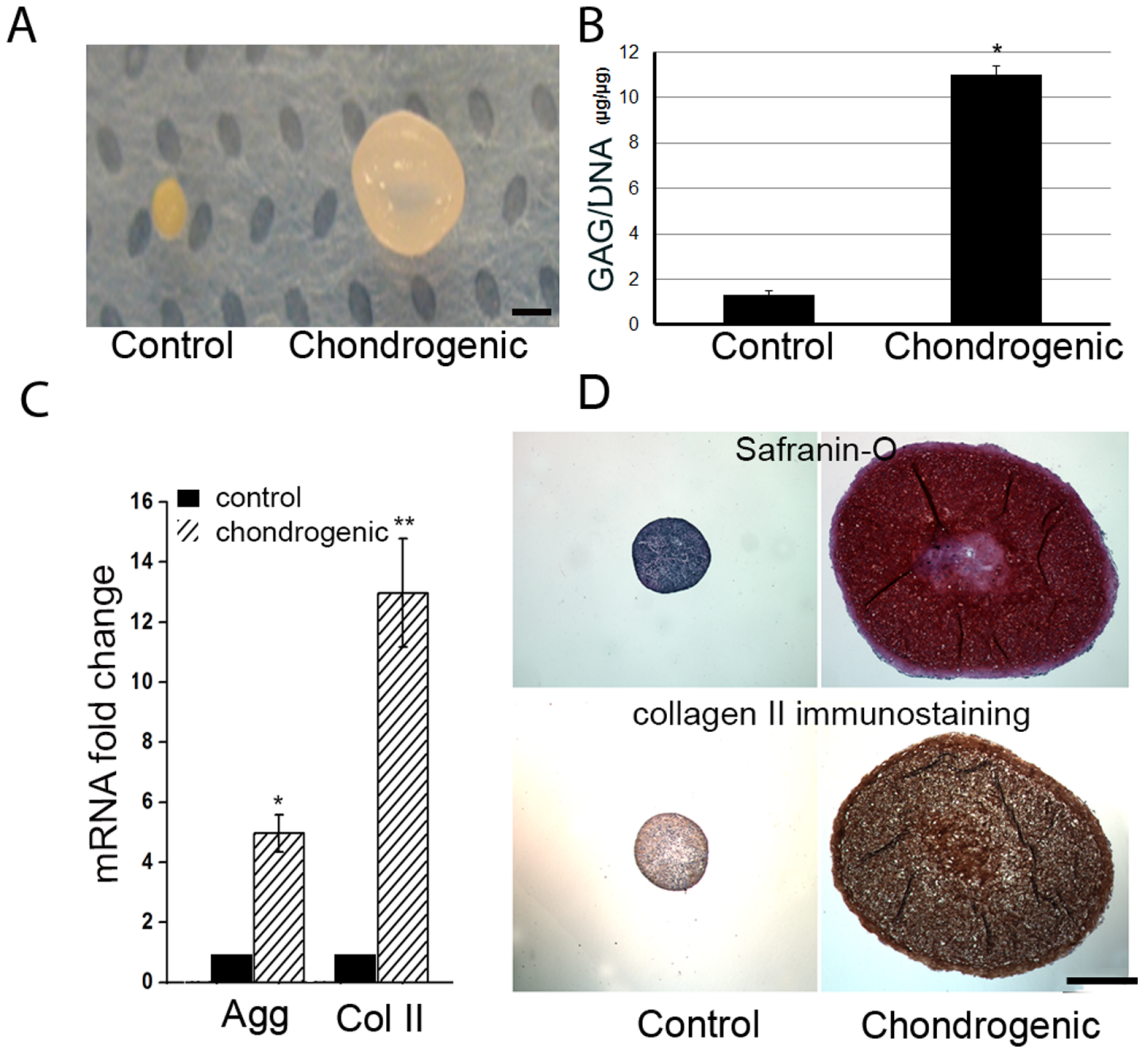
Chondrogenesis of rabbit annulus fibrosus cells *in vitro*. Rabbit annulus fibrosus cells were cultured in a pellet culture system, for three weeks, with chondrogenic medium or control medium (DMEM+1%FBS+1% ITS). A) Gross morphology of cell-pellet. B) GAG in the cell-pellet was measured with the dimethymethylene blue colorimetric assay using chondroitin sulfate as a standard; values are normalized to cell DNA content. C) Expression of collagen II and aggrecan genes are significantly increased in the chondrogenic culture medium compared to control medium. D) Representative images of Safranin-O staining for proteoglycan and immunostaining for type II collagen in the cell-pellet. Scale bar = 500 µm. * p<0.05; ** p<0.01.

We have demonstrated that human AF cells differentiated to various cell lineages and expressed stem cell markers [15]. Chondrogenesis of rabbit AF cells cultured in a 3D condition was confirmed with gene expression, biochemistry, histology, and immunostaining. When treated with chondrogenic medium, the size of cell pellets was much bigger than that of controls (Fig. 1A). mRNA levels of *aggrecan* (5 folds chondrogenic medium vs control medium, *p*<0.05) and *collagen II* (13 folds chondrogenic medium vs control medium, *p*<0.01) of AF cells were significantly up-regulated in chondrogenic medium compared to the controls (Fig. 1C). Consistent with GAG assay (Fig. 1B), Sarfranin-O staining demonstrated a remarkably increased proteoglycan (stained red) in chondrogenic medium-treated pellets, and little red signal presented in the controls (Fig. 1D upper panel). Similarly, immunostaining showed abundant type II collagen protein in the chondrogenic medium-treated pellets (Fig. 1D lower panel).

### Osteogenic Differentiation of Annulus Cells in a Monolayer Culture

**Figure 2:**
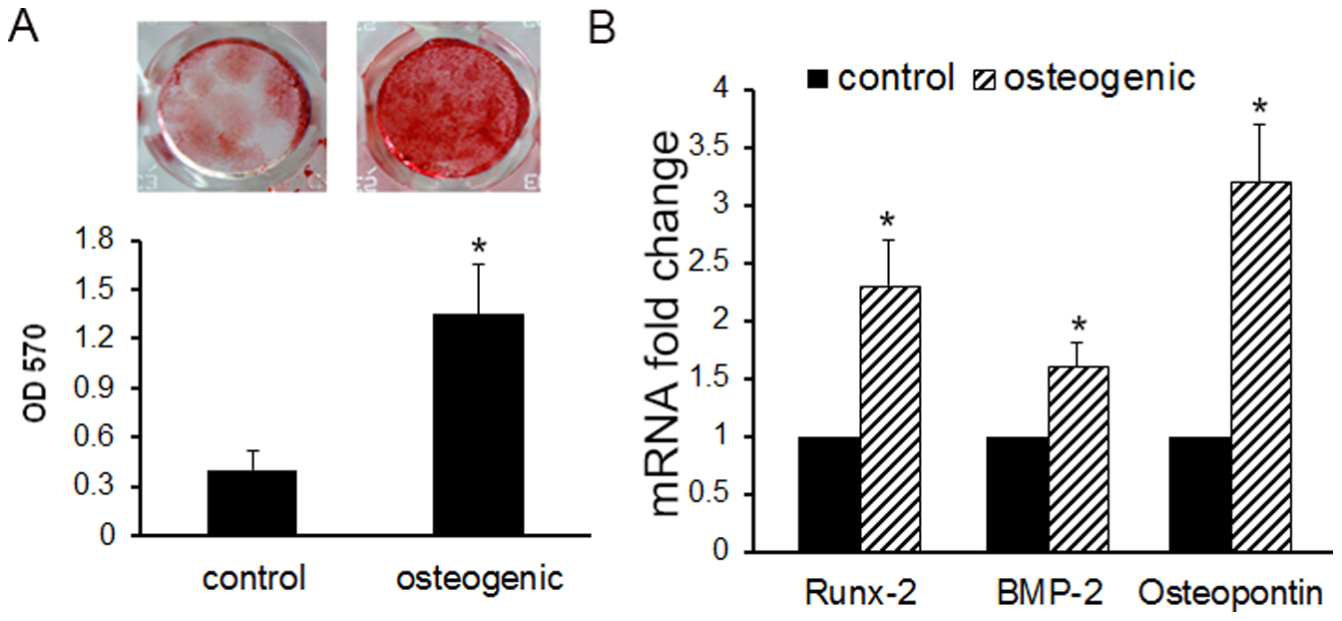
Osteogenesis of rabbit annulus fibrosus cells *in vitro*. Rabbit annulus fibrosus cells were cultured in monolayer, for four weeks, with osteogenic medium or growth medium. A) Alizanin Red staining shows mineralization; the signal was quantified using optical density at 570 nm. B) Expression of Runx-2, BMP-2, and Osteopointin genes markedly increased in cells cultured with osteogenic medium compared to the growth medium. *, p<0.05

The osteogenesis of AF cells in a monolayer culture was also confirmed with gene expression and histology staining. Using Alizarin Red S staining to evaluate the presence of mineralization, AF cells in osteogenic medium exhibited significantly greater calcium deposition than the corresponding control groups (Fig. 2A, 4.5 folds osteogenic medium vs growth medium, *p*<0.05). The expression of *runx2* (runt-related transcription factor 2, a key transcription factor of osteoblast differentiation), *bmp-2* (bone morphogenetic protein 2, an important growth factor in bone and cartilage development), and *osteopontin* were markedly increased in AF cells treated with osteogenic medium compared to that treated with growth medium (Fig. 2B), which indicates the osteogenic potential of these cells.

### Osteogenic Differentiation of Annulus Cell *in vivo*

**Figure 3:**
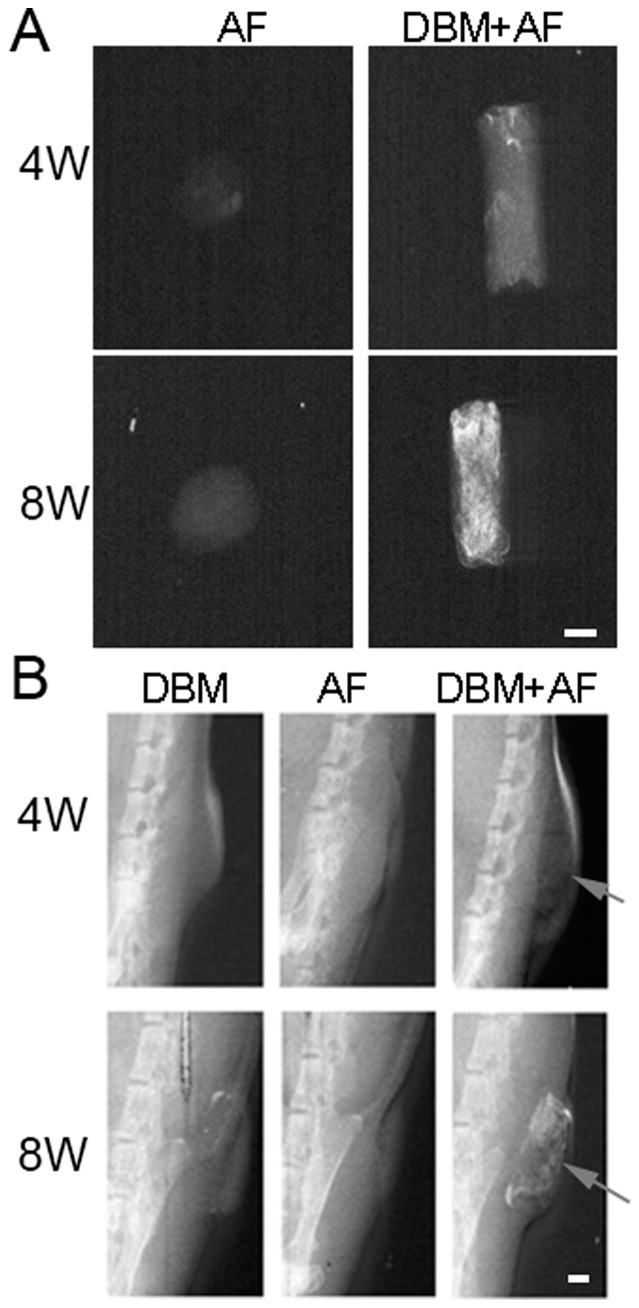
Radiography shows bone formation in DBM/AF constructs that were implanted into the dorsal skin pocket of athymic mice. Rabbit annulus tissue was dissected and fabricated into a DBM cylinder and the constructs were implanted into the dorsal skin pocket of mice. Specimens were harvested at different time points. X-ray images A) in *vitro*, and, B) *in vivo*. Scale bar = 1 mm.

DBM has both osteoinductive and osteoconductive properties [29]. None of the specimens displayed any mineralization at one week after implantation, however, by week 4, DBM/AF constructs began to demonstrate bone formation as determined radiographically *in vitro* (Fig. 3 A, white signal). By week 8, the degree of mineralization increased significantly and was visualized both *in vitro* and *in vivo* with x-ray evaluation (Fig. 3 B). In contrast, the AF by itself showed no sign of mineralization over the course of this study. DBM by itself showed mild ossification at week 8. We also inserted fat tissue into DBM to serve as a control. Up to 12 weeks, we did not see significant bone formation compared with the DBM group (data not shown).

**Figure 4:**
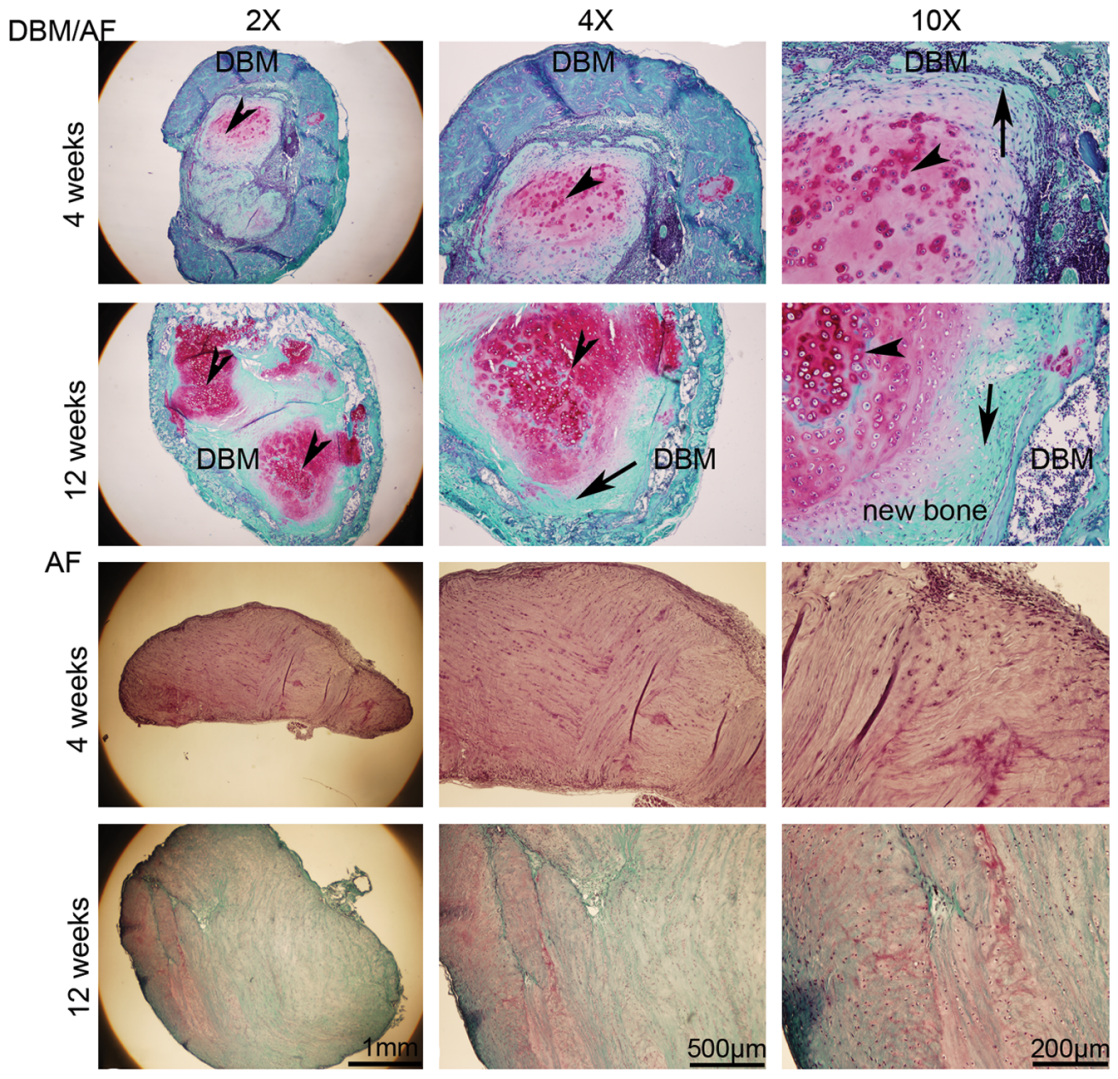
Safranin-O staining of subcutaneously implanted DBM/AF constructs. Specimens were harvested at 4 and 12 weeks postoperatively and embedded in paraffin. Sections, 7 µm thick, were stained with Safranin-O. Red colors denote proteoglycan signal (arrow head). Osteoblasts appeared at the junction of DBM and AF tissues (arrows).

**Figure 5:**
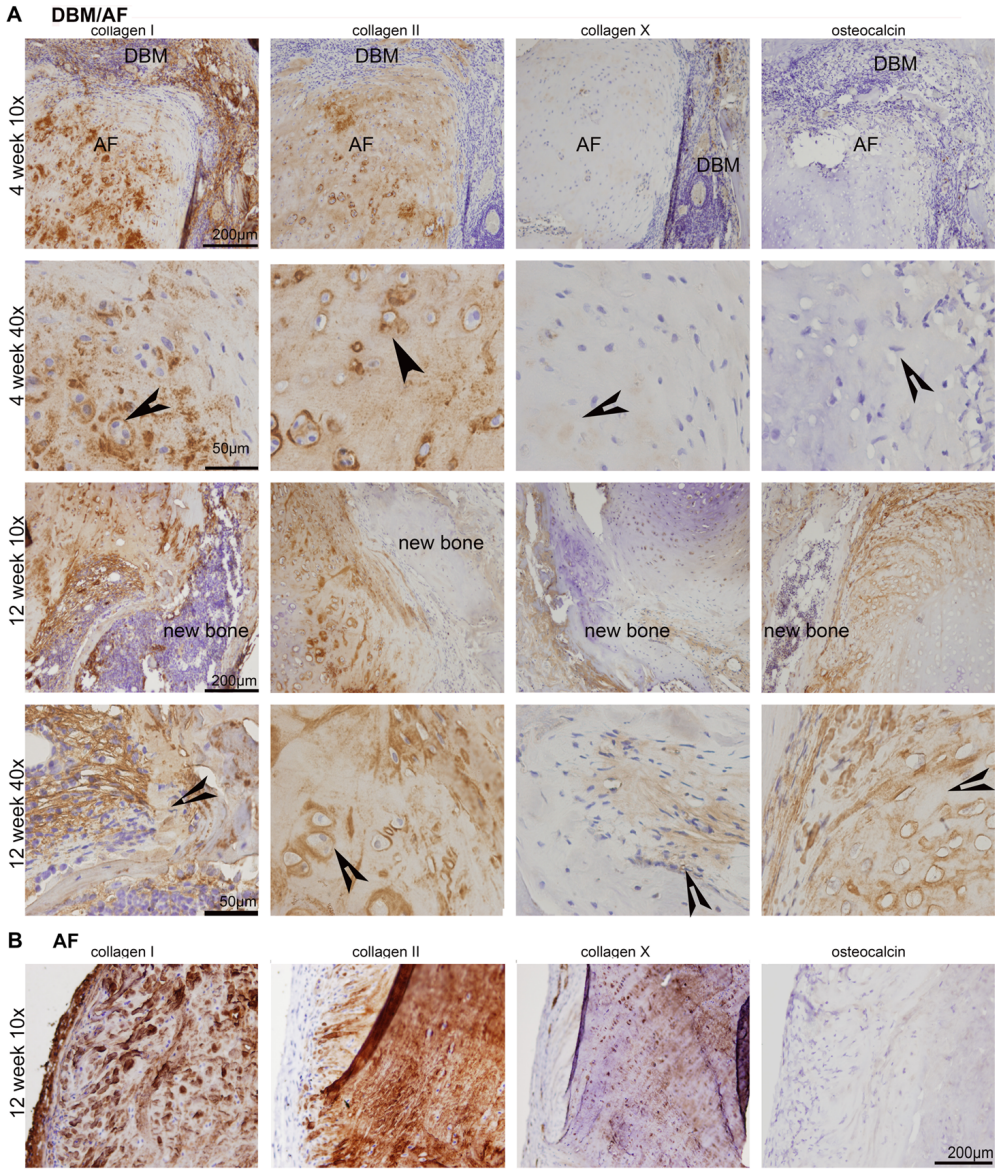
Immunohistochemitry detection of subcutaneously implanted DBM/AF (A) or AF alone constructs (B). Separate sections were treated with collagen I, collagen II, collagen X, and osteocalcin primary antibodies and corresponding secondary biotinylated IgG. The signals were developed with 3,3′-diaminobenzidine and photographed on an Axioskop 2 Zeiss microscope. Five sections from four discs were used at each time point. Arrows showing hypertrophy chondrocytes.

Safranin-O staining clearly demonstrated that AF cells differentiated into chondrocytes and osteoblasts in a bony environment, and that AF tissue integrated well with the outside DBM cylinder (Fig. 4). Red signal denotes pronounced proteoglycan surrounding hypertrophic chondrocytes (arrow head). Osteoblasts appeared at the junction of DBM and AF tissues (arrows). AF only implants showed no signal of cell differentiation (Fig. 4 lower panels). DBM constructs showed some fibrotic tissue in the center with no further change in the DBM ring (data not shown). Immunohistochemistry at week 4, collagen I showed in both AF and DBM tissues, while collagen II was largely expressed in AF tissue. Some collagen X signal appeared at the junction of AF tissue and the DBM ring of the construct. At week 12, significant signal for collagen X and osteocalcin were seen at the junction of DBM and AF tissues (Fig. 5A). However, in AF tissue without DBM collagen I and collagen II were abundantly expressed, and the faint signal of collagen X was detected (Fig. 5B).

### Chondrogenic and Osteogenic Phenotype in Injured Discs

**Figure 6:**
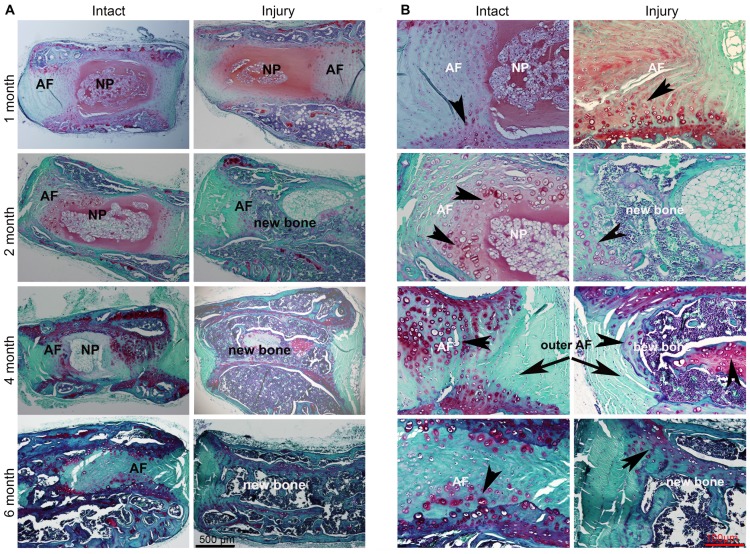
Hypertrophic chondrocytes and osteoblasts in punctured intervertebral disc specimens with Safranin-O staining. The rat intervertebral discs including endplates, NP, and AF tissues were punctured with a needle and implanted into the dorsal skin pocket of athymic mice. The specimens were harvested at 1, 2, 4, and 6 months postoperatively. Tissue sections stained with Safranin-O for disc structure and constituent proteoglycan staining shown in red (A, 4 x magnification; B,10 x magnification). Arrow: hypertrophy chondrocytes. Five sections from four discs were used at each time point.

**Figure 7:**
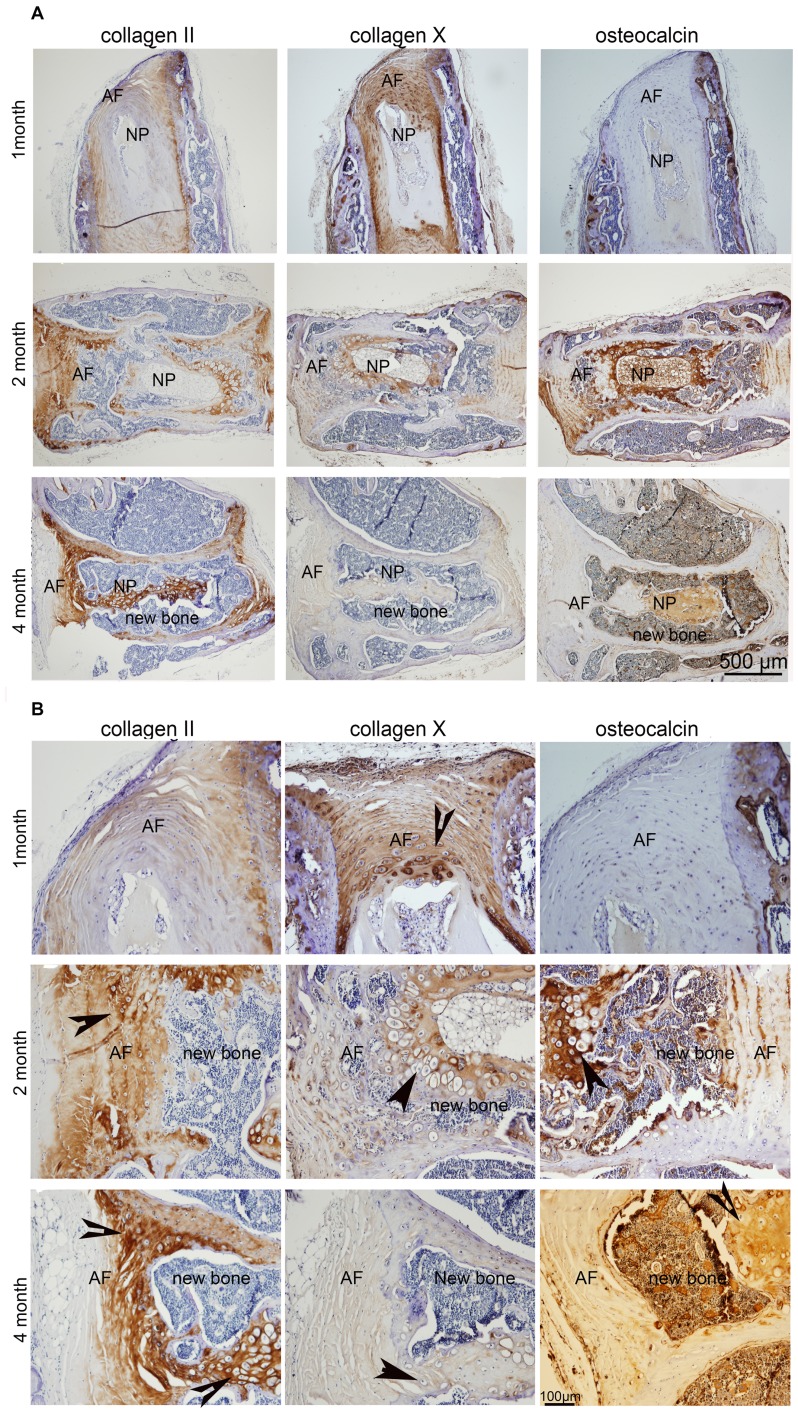
Immunohistochemistry shows the location and distribution of collagens and osteocalcin in punctured intervertebral discs at the different postoperative time points. Brown signal shown the positive staining of the targeted protein (A, 4 x magnification; B, 10 x magnification). Arrow denotes hypertrophy chondrocytes. Five sections from four discs were used at each time point.

We further investigated the differentiation potential of AF cells in a simulated disc herniation model by puncturing the disc with a 27G needle. One month after implantation, the hypertrophic chondrocytes (arrow head) were seen in the inner AF region by Safranin-O staining. Two months after implantation, more inner AF tissue differentiated to hypertrophic cartilage-like tissue and bone tissue (Fig. 6 A&B). In the uninjured discs up to 2 months, the NP and AF tissue were clearly seen, and only small amounts of bone had formed at 4 months. At 6 months, almost all the disc area became bone tissue in the injured discs except for the outer layer of AF (arrow). Immunohistochemical analysis confirmed the same changes. At 1 month, in contrast to the faint osteocalcin signal, collagen II and X were abundant in the AF area. At 2 months, collagen X was dominant in the surrounding area of NP/AF and the inner AF. However, a significant amount of osteocalcin was observed in the NP/AF area. Towards 4 months after implantation, more bone tissue occupied the disc area with dissipating collagen X, while osteocalcin was widely expressed (Fig. 7 A&B).

## Discussion

IVD degeneration has been extensively studied, but the precise etiology and pathophysiology of disc disease remains to be explored. Various factors have been suggested to influence the etiology of degeneration including biomechanical factors [30], such as compressive loading, shear stress and vibration. Other potential contributors are aging [31], genetic [8], [32], systemic, and toxic factors, which lead to degeneration of the disc through biochemical reactions [33]. Disc degeneration will occur if the anabolic and catabolic metabolism of the matrix is not well balanced. This can arise if synthesized components are themselves abnormal, or if the balance between synthesis and degradation of normal components is disturbed in favor of degradation.

Studying progenitor cells will aid our understanding of how they transform into the specialized cells. Some of the most serious medical conditions, such as cancer, aging, and birth defects, are due to problems that occur somewhere in the process of stem cell differentiation. Stem cells have been suggested as the genesis of cancer [34], while the body of work regarding stem cells and aging is steadily increasing. A better understanding of normal cell development will allow us to understand and perhaps correct the errors that cause these medical conditions. Thus far, scientists have found stem cells in several mesoderm-derived tissues, including bone marrow, fat, skin, dura mater, and tendons [15], [35], [36]. Interestingly, while the NP is considered to be a remnant of the notochord, the AF is thought to be derived from mesoderm. The growth of fibrocartilage-like tissue, bone, neural tissue, and blood vessels in IVDs during degeneration [11], [37] is suggestive of the presence of progenitor cells that either derived from surrounding tissue, or are present within the IVDs. Using a rabbit disc degeneration model, we previously demonstrated that injection of a 30 kDa fragment of fibronectin into the AF led to the formation of osteophytes in the anterior region of the AF, a peripheral region of cartilaginous tissue with a central region of osteoid and cancellous bone [38]. In addition, abnormal innervation, which occurs when pain is associated with IVD degeneration, has been linked to the production of nerve growth factor (NGF) in the degenerative IVD [39]. Masuda *et al*. found that human AF and NP cells constitutively express both NGF protein and mRNA, and the proinflammatory cytokines IL-1β and TNF-α stimulate the production of NGF [40].

The normal IVD is an avascular and aneural tissue [41]. Nutrients and metabolites are exchanged via capillaries at the endplate interface by diffusion. The mechanisms involved in pathological innervation and vascularization of the IVD are not yet understood. These processes are currently considered to be resulting from the migration of cells from outside of the IVD [11], [42]. In the degenerated discs, a large number of growth factors such as FGF, TGF-β1, NGF, and CTGF (connective tissue growth factor) are increased in the local discs. These growth factors may attract the cells that migrate from the outer lamellae of the AF, longitudinal ligament of discs, and an area adjacent to the epiphyseal plate [13], [14], [18]. Many studies suggest that neovascularization and nerve formation occur in the degenerated discs, but there is no direct evidence to support vessel and nerve ingrowth from outside the tissue. On the other hand, these growth factors may also stimulate the differentiation of cells that reside within the disc. Using an alginate beads culture system, Chelberg *et al*. have provided evidence that more than one distinctive cell population resides in the human disc [43]. In our previous study, we found that AF cells isolated from normal human IVDs differentiated into different lineages under corresponding stimuli [15]. Moreover, others also reported that disc cells have differentiation capabilities [16], [21]. Hegewald *et al*. reported that human degenerated lumbar AF cells expressed type X collagen when treated with TGF-β3 [44]. Kandel's group demonstrated that a sub-population of healthy AF cells has mineralization potential and AF cells developed toward an osteogenic-phenotype under appropriate conditions [45]. We hypothesize that the ectopic bone formation, innervation, and vascularization in the degenerative IVD is also a result of progenitor cell differentiation in the inner AF tissue because biomechanical and/or biochemical alterations change the AF progenitor cell niche, leading to differentiation of indwelling cells and disc degeneration. Consistent with others [18], [19], [46] and our previous finding in human AF cells [15], we show here that the rabbit AF cells have the capacity to differentiate into osteoblasts and chondrocytes under appropriate culture conditions in a fashion similar to human AF cells (Fig. 1&2). In a 3D pellet culture system, AF cells express more type II collagen and proteoglycan, both at the mRNA and protein levels, in the chondrogenic media culture compared to the cells cultured in the control medium. In the osteogenic condition, both *runx-2* and *bmp2* genes, an important transcription factor and a bone formation factor, respectively, were significantly up-regulated. Similarly, osteopontin increased markedly in osteogenic media compared to the growth media controls. These results suggest that the pluripotency of AF cells is conserved across species.

However, in vitro only experimentation does not answer the question of the origin of the pluripotency, whether it comes from, differentiation or dedifferentiation? Some cells could dediffentiate into stem cells [47], i.e. luminal secretory cells [48], cardiomyocytes [49], blastema cells [50], and Schwann cells [51]. Many differentiated cells, i.e. hepatocytes and articular cartilage chondrocytes, have been demonstrated dedifferentiation in a monolayer culture [52], [53]. Similarly both AF and NP cells have been shown to undergo morphological and biochemical changes during primary monolayer culture [54]. However, in a three-dimensional culture model, Gruber *et al*. [55] demonstrated that the production of collagen I, II, 4-sulfated chondroitin sulfate, and keratan sulfate were stable through multiple passages and support the phenotypic stability of disc cells in a three dimensional culture. In the present study, the AF cells were cultured in 3D with chondrogenic media or control media, and the results showed that chondrogenic medium was conducive to AF cell differentiation into chondrocytes. This result is in line with other reports [18]-[20]. Therefore, it is unlikely that the chondrogenic differentiation we observed resulted from dedifferentiation, which is known to take place in monolayer cultures.

In order to further characterize the pluripotency, we performed two *in vivo* experiments: AF fabricated with DBM, and injured disc organ. Experimental ectopic bone formation has been studied in the muscle pouch [56], [57], subcutaneous [58], [59], and in kidney capsule models [60]. The advantages of the ectopic model over the anatomical bone environment is that it ignores the biochemical and mechanical factors of bone formation cascades, deprives the immunorejection of immunodeficiency rodents, and removes the extraneous variables such as cytokines, growth factors, and mechano-transduction pathways [61]. Subcutaneous implantation is a straightforward and low-cost method to study the ectopic bone formation. When the DBM/AF constructs were subcutaneously implanted in the dorsal pockets of nude mouse, bone formation was observed as early as 4 weeks and intensified by week 8 as detected by radiography (Fig. 3). In contrast, little bone formation was seen in either AF or DBM implants alone. Consistent with the Safranin-O staining, the expression of collagen X and osteocalcin in addition to collagen I and II was detected in the DBM/AF constructs by immunohistochemistry (Fig. 5 a and b). These data support two theories: first, that the decalcified bone matrix provides a bone-promoting environment; second, native AF tissues are able to differentiate to hypertrophic chondrocytes and osteoblasts in an appropriate biochemical bone formation stimuli. In the other *in vivo* model, when the injured rat intervertebral discs were implanted subcutaneously, the discs sustained two kinds of biomechanical environmental changes. A loss of physiologic loading occurred upon removal, and a break in the sealed structure was the result of needle puncture that mimicked disc herniation. Subsequent to the above biomechanical stimuli, hypertrophic chondrocytes (arrow head) and osteoblasts were present in the disc 1 month after the operation (Fig. 6 & 7). The increased bone formation was found to be proportional to the length of time after implantation. For the intact disc implantation, osteoblasts appeared in the disc area at 4 months but the condition was less severe at 6 months. However, by 8 months, most of the injured disc tissue became bone (data not shown). These data strongly support our conclusions that disc cells and disc tissue develop osteogenesis both *in vitro* and *in vivo*.

The current study still has two shortcomings. One, we still could not exclude the possibility of a migration of the cells from the adjacent tissue. However, the disc was punctured through the outer annulus to inner annulus. The differentiation into cartilage-like or bone tissue takes place in the NP and inner AF region, but not in the outer AF region. We would argue that if there is a migration of cells from the adjacent tissue, the outer AF should have more tissue type change. However, up to 8 weeks, outer AF did not show significant sign of differentiation. Second, we could not claim there are stem cells in the inner AF tissue as we did not perform sufficient stem cell characterization, which include clongenicity and self-renewal, in addition to multipotency, which was investigated here. The findings of this study extend our knowledge on AF cell biology that a subset of disc cells is capable of differentiating into different lineage cells upon appropriate stimuli. The characterization of these progenitor cells may provide a new tool for the study of IVD biology and build a foundation for potential future therapies that include the targeting of progenitor cell stabilization or by maintaining the capability for undifferentiated self-renewal.
